# A case report of delayed onset of severe aortic regurgitation due to commissural detachment after low-energy blunt chest trauma: timing is key

**DOI:** 10.1093/ehjcr/ytaf446

**Published:** 2025-09-16

**Authors:** Emiyo Sugiura, Masaki Ishiyama, Hisato Ito, Shiro Nakamori, Kaoru Dohi

**Affiliations:** Department of Cardiology and Nephrology, Mie University Graduate School of Medicine, 2-174 Edobashi, Tsu, Mie 514-8507, Japan; Department of Cardiology and Nephrology, Mie University Graduate School of Medicine, 2-174 Edobashi, Tsu, Mie 514-8507, Japan; Department of Thoracic and Cardiovascular Surgery, Mie University Hospital, 2-174 Edobashi, Tsu, Mie 514-8507, Japan; Department of Cardiology and Nephrology, Mie University Graduate School of Medicine, 2-174 Edobashi, Tsu, Mie 514-8507, Japan; Department of Cardiology and Nephrology, Mie University Graduate School of Medicine, 2-174 Edobashi, Tsu, Mie 514-8507, Japan

**Keywords:** Aortic regurgitation, Blunt chest trauma, Delayed onset, Commissural detachment, Valve replacement, Case report

## Abstract

**Background:**

Valve injury is a rare complication of blunt chest trauma (BCT). Although many cases result from high-energy BCT, it is important to recognize that low-energy trauma can also lead to serious cardiac complications, depending on the timing of the applied force. Herein, we present a case of delayed traumatic aortic regurgitation (AR) following low-energy BCT.

**Case summary:**

A 77-year-old man presented with acute heart failure due to severe AR 2 months after a minor fall. Echocardiography revealed commissural detachment between the left and noncoronary cusps (NCC). He underwent aortic valve replacement owing to uncontrolled heart failure and severe AR.

**Discussion:**

This case demonstrates delayed traumatic AR following low-energy BCT, a presentation less common than its typical association with high-energy trauma. However, AR can occur after minor trauma, particularly if the impact takes place during early diastole. The NCC is most frequently involved. In this case, detachment occurred at the commissure between the NCC and the left coronary cusp. Patients with acute and severe AR require prompt surgical intervention. This case emphasizes the importance of considering follow-up echocardiography in patients with persistent or progressive cardiac symptoms, even after minor chest trauma.

Learning points

- Valvular injury is a rare complication of blunt chest trauma (BCT).
- Although most cases of traumatic aortic regurgitation (AR) result from high-energy BCT, it is important to recognize that even low-energy trauma can lead to serious cardiac complications, particularly when the impact occurs during a vulnerable phase of the cardiac cycle.
- Traumatic AR may develop in a delayed fashion due to the progressive deterioration of microstructural damage caused by initial trauma and sustained haemodynamic stress.

## Introduction

Traumatic aortic regurgitation (AR) is a rare but potentially fatal consequence of blunt chest trauma (BCT). It most commonly occurs acutely and is associated with high-energy trauma. However, delayed-onset AR has also been reported, often resulting from progressive structural damage. We report a rare case of delayed severe AR following a minor fall, in which the patient initially showed no clinical signs of valvular dysfunction. This case illustrates how even low-energy trauma, when occurring during a vulnerable phase of the cardiac cycle, may lead to severe valvular injury.

## Summary figure

**Figure:**
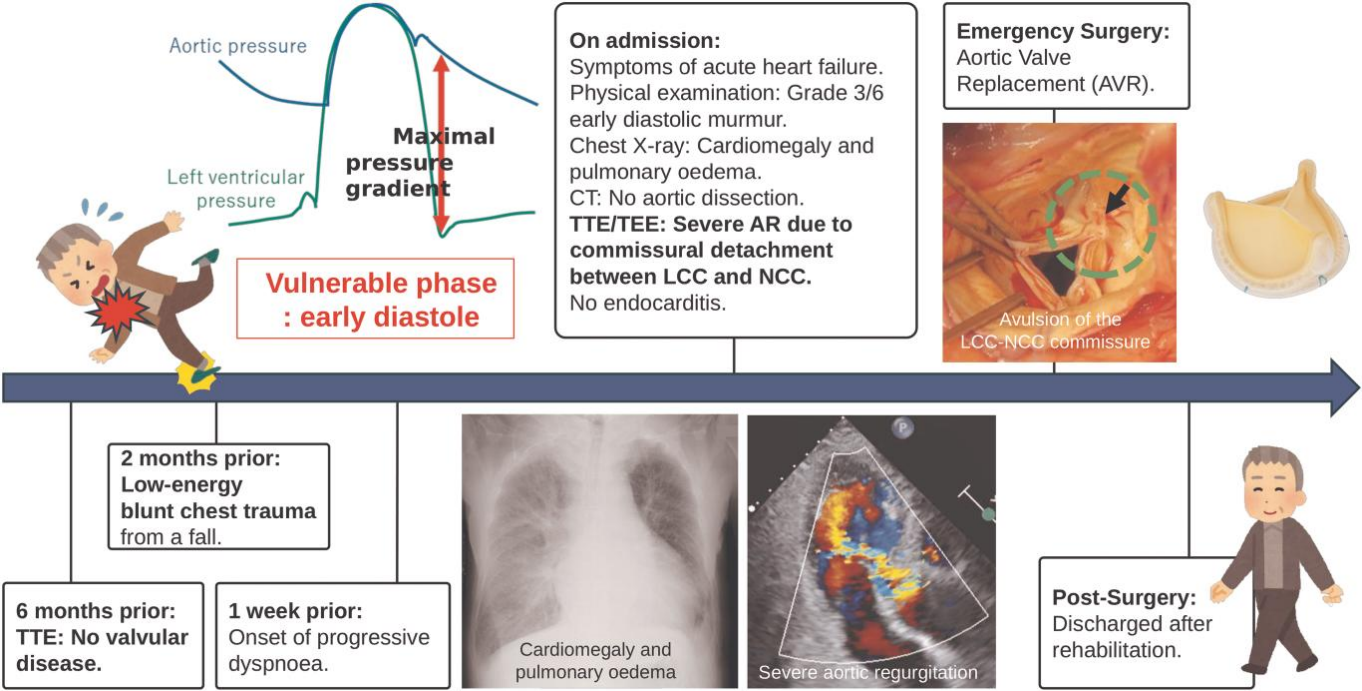


## Case presentation

A 77-year-old man presented with acute heart failure. His medical history included hypertension and recent oncologic surgeries; a low anterior resection for rectal cancer 8 months prior and a transurethral resection for a bladder tumour 6 months earlier. Notably, transthoracic echocardiography (TTE) performed 6 months before admission revealed no valvular abnormalities.

Two months prior to admission, the patient sustained BCT from a standing fall but did not seek medical attention due to mild symptoms. One week before admission, he developed dyspnoea and was diagnosed with congestive heart failure at a local hospital, leading to referral to our facility.

On admission, his vital signs were: blood pressure 150/50 mmHg; heart rate, 110 bpm; respiratory rate, 22 breaths/min; and oxygen saturation, 95% on 6 L/min of oxygen via a reservoir mask. Physical examination revealed jugular venous distension and a grade 3/6 early diastolic murmur best heard at the left sternal border. Bilateral coarse crackles were noted on lung auscultation; however, no peripheral oedema was observed.

**Figure 1:**
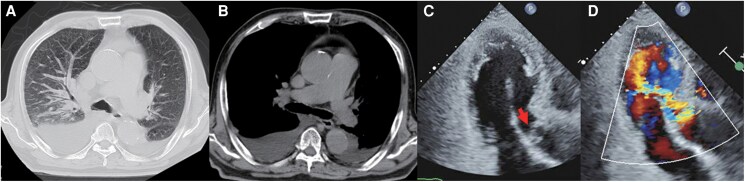
Computed tomography and transthoracic echocardiography findings. Chest computed tomography findings included pulmonary oedema, bilateral pleural effusion (*A*), and mild ascending aortic dilatation without evidence of aortic dissection (*B*). (*C*) Apical long-axis view on transthoracic echocardiography revealing aortic valve prolapse (arrow). (*D*) Colour Doppler imaging on transthoracic echocardiography demonstrating severe aortic regurgitation with an eccentric jet. CT, computed tomography; TTE, transthoracic echocardiography; AR, aortic regurgitation.

Laboratory results showed: brain natriuretic peptide, 1742 pg/mL; white blood cell count, 13 870/μL; C-reactive protein, 2.51 mg/dL; and estimated glomerular filtration rate, 35.0 mL/min/1.73 m^2^. Chest radiography showed cardiomegaly (cardiothoracic ratio, 62%), pulmonary oedema, and bilateral pleural effusions. Computed tomography (CT) ruled out aortic dissection but revealed mild dilation of the ascending aorta (42 mm) (*Figure 1A* and *B*).

Transthoracic echocardiography demonstrated a preserved left ventricular ejection fraction (70%), left ventricular end-diastolic diameter (57 mm), and end-systolic diameter (33 mm). The left atrial volume index was 38 mL/m² and the inferior vena cava was dilated (28 mm) with reduced respiratory variation. Severe AR was noted (*Figure 1C* and *D*, Supplementary material online, *Videos S1* and *S2*), with an effective regurgitant orifice area of 0.57 cm^2^, pressure half-time of 175 ms, and a vena contracta width of 7 mm.

**Figure 2:**
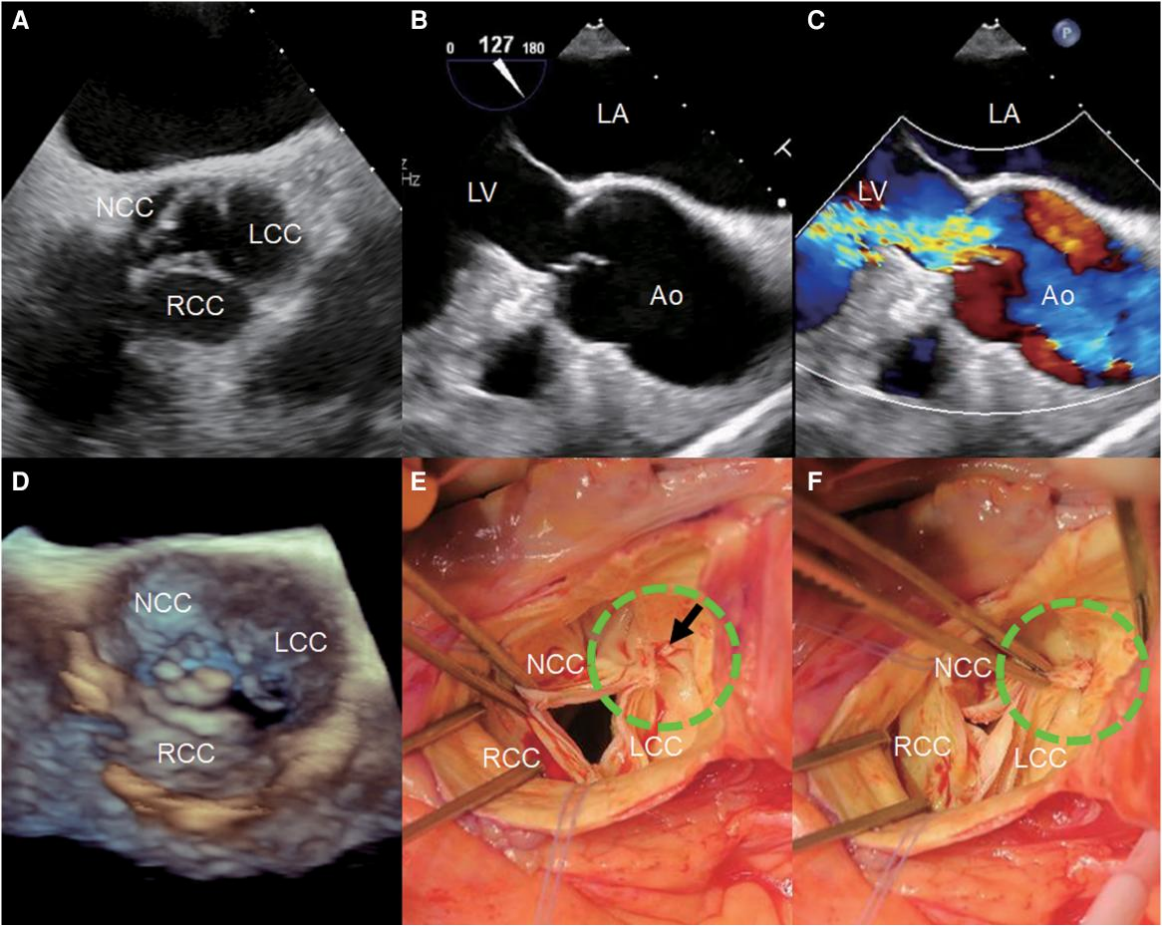
Transoesophageal echocardiography and intraoperative findings. (*A*) Transoesophageal echocardiography short-axis view of the aortic valve. (*B*) Transoesophageal echocardiography long-axis view showing aortic valve prolapse into the left ventricle. (*C*) Colour Doppler transoesophageal echocardiography long-axis view demonstrating severe AR with a broad jet. (*D*) 3D-en-face transoesophageal echocardiography view clearly showing prolapse of the left coronary cusp and noncoronary cusp. Intraoperative images from the aortic side revealed a tear (arrow) in the intima at the commissure between the left coronary cusp and noncoronary cusp (*E*), confirming commissural detachment (*F*). NCC, noncoronary cusp; RCC, right coronary cusp; LCC, left coronary cusp; LV, left ventricle; LA, left atrium; Ao, aorta; TEE, transoesophageal echocardiography.

Transoesophageal echocardiography (TEE) confirmed severe AR due to commissural detachment between the left coronary cusp (LCC) and the noncoronary cusp (NCC) (*Figure 2A–D*, Supplementary material online, *Videos S3–S6*). No vegetations or features suggestive of infective endocarditis were observed, and blood cultures were negative. Cardiac magnetic resonance imaging was not performed due to the patient’s unstable respiratory status, and sufficient diagnostic and treatment planning information was obtained from echocardiography and CT.

Despite optimal medical therapy, the patient’s condition deteriorated, prompting urgent surgical aortic valve replacement (AVR). Intraoperative findings included avulsion at the LCC-NCC commissure without leaflet perforation or endocarditis (*Figure 2E* and *F*). A 25-mm Avalus bioprosthetic valve was implanted, and the commissure was reattached. The postoperative course was uneventful. Histopathological examination of the excised valves showed no evidence of infection. Postoperatively, the patient developed muscle weakness, and was transferred to another hospital for rehabilitation. He was discharged following completion of rehabilitation and has remained in stable condition for 6 months since discharge.

## Discussion

This case illustrates a rare presentation of delayed-onset traumatic AR following low-energy BCT. While significant traumatic AR is typically associated with high-energy mechanisms such as motor vehicle accidents or falls from a height, this case highlights that even minor trauma can result in severe valvular injury, particularly if it occurs during a vulnerable phase of the cardiac cycle.

The exact timing of traumatic AR onset remains debated. Some reports suggest that it occurs during early systole, when left ventricular pressure is at its highest.^1^ However, the prevailing view favours early diastole. During early diastole, the aortic valve is closed, and the maximal pressure gradient across the valve generates a force that pushes the aortic valve leaflets towards the left ventricle. A sudden increase in intra-aortic pressure caused by BCT leads to a phenomenon known as the ‘water hammer’ effect.^2^

Conversely, if an abrupt external force is applied during systole, it may elevate intraventricular pressure and result in pressure-related injuries to the mitral valve and aorta. However, because the aortic valve is open during systole, the pressure gradient across it is minimized, thereby reducing the likelihood of direct injury to the aortic valve cusps.

In the present case, LCC-NCC commissural detachment was observed, suggesting that external force may have displaced the aortic valve towards the left ventricle and indicated a pressure-related injury occurring during diastole.

Regarding the site of injury, the NCC is the most frequently affected site. This is likely because, unlike the right coronary cusp (RCC) and LCC, it lacks a coronary ostium. Due to its anterior location, the RCC is the second most damaged site after the NCC, while the LCC is the least frequently injured.^3^ In our patient, injury occurred at the commissure between the NCC and LCC, leading to cusp prolapse and severe regurgitation. Isolated commissural detachment without cusp rupture is rare, but a recognized possibility.

A key feature in this case was the delayed onset of severe AR, suggesting a progressive mechanism rather than immediate acute decompensation. Although traumatic AR may present acutely, the delayed symptoms in our patient likely stemmed from a subclinical initial injury to the aortic valve apparatus that went unrecognized immediately after trauma. Subclinical lesions, such as microtears, isolated commissural disinsertion, or structural weakening may initially be asymptomatic but are subjected to cumulative haemodynamic stress from the ongoing ‘water hammer effect’, which repeatedly burdens the compromised valve.^3,4^ This continuous mechanical strain on an inherently or subtly weakened structure eventually may culminate in delayed structural decompensation and progressive AR.

Contributing factors such as age-related connective tissue vulnerability or pre-existing predispositions may also influence the extent and progression of such an initial injury. For instance, inherent tissue fragility, as observed in conditions such as Marfan syndrome, can cause a small tear to extend rapidly under ongoing haemodynamic stress.^5^ Although histopathological examination of the valve in this case did not reveal features such as fibrosis or myxoid changes, their presence in other cases would further support a progressive injury mechanism. This gradual worsening, due to haemodynamic stress or degeneration changes, often leads to symptom onset weeks or even months after the initial trauma, as observed in our patient.

Najafi *et al*.^6^ proposed diagnostic criteria for traumatic AR, which include: (i) a clear history of trauma, (ii) absence of pre-existing valvular disease, (iii) sudden or progressive onset of AR post-injury, and (iv) exclusion of other aetiologies such as endocarditis. Our case met all of these criteria. Negative blood cultures and the absence of vegetations or inflammatory signs effectively ruled out infective endocarditis.

Echocardiography is the cornerstone of diagnosis. Transthoracic echocardiography is useful for assessing AR severity and detecting cusp prolapse or flail, while TEE provides superior visualization of valve morphology, particularly for identifying commissural tears, leaflet avulsions, or perforations.^7^ In our patient, TEE was pivotal in precisely locating the valvular disruption. Eccentric AR jets may be underestimated on TEE due to colour Doppler visualization, underscoring the importance of TTE in accurate assessment. In select cases, cardiac catheterization or aortography may provide further diagnostic clarity, and CT is useful for excluding associated injuries, such as aortic dissection.

Surgical intervention is typically required for patients with severe, symptomatic traumatic AR. According to the 2021 ESC/EACTS guidelines, early surgery is indicated in patients with severe AR and heart failure symptoms.^8^ Our patient underwent a timely surgical intervention, which resulted in a favourable outcome.

The choice between AVR and aortic valve repair (AVP) depends on injury characteristics, patient factors (age, comorbidities), and surgical expertise. Aortic valve replacement remains the standard approach for complex or extensive injuries, and offers reliable outcomes. In contrast, AVP may be considered in younger patients or in those with isolated, repairable lesions, as it preserves the native valve and avoids anticoagulation.^9^ However, the long-term durability of repair remains a concern.^10^ Intraoperative TEE is essential to evaluate the adequacy of repair. For this patient, who was in his late 70s, AVR was chosen over AVP. This decision was based on a comprehensive assessment of several factors, including age-related tissue fragility and the critical need to minimize operative time, ensure long-term valve durability, and achieve procedural certainty. Although J-Valve transcatheter aortic valve implantation was considered a potential alternative, surgical AVR was ultimately selected given the patient's eligibility for surgical intervention and the device’s current unapproved status in Japan. The timing of surgery can vary; it may be emergent in unstable patients or delayed when the clinical status permits. Some patients remain asymptomatic for days or longer, necessitating careful monitoring for signs of sudden decompensation.

Traumatic AR may occasionally coexist with other valvular injuries, particularly mitral valve damage. However, isolated aortic valve involvement is common. Histopathological analysis can reveal subtle changes that are not visible intraoperatively, further confirming the traumatic nature of the injury.

Traumatic AR represents a rare but clinically significant complication of BCT. Even low-energy trauma can result in severe AR; thus, a high index of suspicion is warranted in patients with new murmurs or unexplained heart failure following chest trauma. Echocardiography is indispensable for diagnosis and surgical planning. Prompt intervention guided by the current guidelines can result in excellent outcomes. Long-term follow-up with echocardiographic reassessment of new symptoms is essential.

## Lead author biography


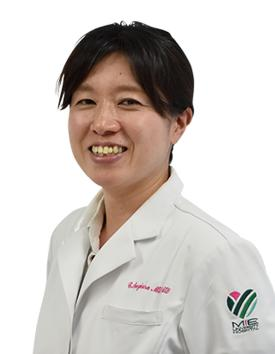


Dr Sugiura received her medical degree and completed her specialization in cardiovascular medicine at the Mie University Graduate School of Medicine in Japan. She is currently working as an echocardiologist at Mie University. Her areas of interest include echocardiography and structural heart interventions.

## Supplementary Material

ytaf446_Supplementary_Data

## Data Availability

The data underlying this article will be shared at reasonable request to the corresponding author.

## References

[1] Parmley LF, Mattingly TW, Manion WC, Jahnke EJ Jr. Nonpenetrating traumatic injury of the aorta. Circulation 1958;17:1086–1101. 13547374 10.1161/01.cir.17.6.1086

[2] de Manna ND, Hoxha S, Cerrito LF, Monica C, Faggian G, Luciani GB. Late aortic valve rupture after blunt chest trauma. Heart Lung Circ 2020;29:e279–e280. 32718899 10.1016/j.hlc.2020.06.001

[3] Li W, Ni Y, Chen X, Ma L. Aortic valve tear with severe aortic regurgitation following blunt chest trauma. J Cardiothorac Surg 2011;6:84. 21682925 10.1186/1749-8090-6-84 PMC3133542

[4] Pretre R, Chilcott M. Blunt trauma to the heart and great vessels. N Engl J Med 1997;336:626–632. 9032049 10.1056/NEJM199702273360906

[5] Miyashita N, Onoe M, Nakamoto S, Satsu T, Fujii K, Nishino T, et al Aortic valve repair of traumatic aortic regurgitation to a young woman. Jpn J Cardiovasc Surg 2017;46:6–10.

[6] Najafi H, Dye WS, Javid H, Hunter JA, Goldin MD, Serry C. Traumatic aortic valve insufficiency. J Thorac Cardiovasc Surg 1979;77:755–760.

[7] Chirillo F, Totis O, Cavarzerani A, Meneghetti G, Casarotto D, Olivotto G, et al Usefulness of transthoracic and transoesophageal echocardiography in recognition and management of cardiovascular injuries after blunt chest trauma. Heart 1996;75:301–306. 8800997 10.1136/hrt.75.3.301 PMC484291

[8] Vahanian A, Beyersdorf F, Praz F, Milojevic M, Baldus S, Bauersachs J, et al 2021 ESC/EACTS guidelines for the management of valvular heart disease. Eur Heart J 2022;43:561–632. 34453165 10.1093/eurheartj/ehab395

[9] Sassis L, Kefala-Karli P, Cucchi I, Kouremenos I, Demosthenous M, Diplaris K. Valve repair in aortic insufficiency: a state-of-the-art review. Curr Cardiol Rev 2023;19:e270422204131. 35490315 10.2174/1573403X18666220427120235 PMC10201877

[10] Filsoufi F, Salzberg SP, Adams DH. Current results of aortic valve repair. Curr Opin Cardiol 2005;20:120–124. 10.1097/01.hco.0000179817.88200.60 16234622

